# Combined assessment of serum folate and hemoglobin as biomarkers of brain amyloid β accumulation

**DOI:** 10.1371/journal.pone.0175854

**Published:** 2017-04-13

**Authors:** Takuma Yoshinaga, Hiroto Nishimata, Yoriko Kajiya, Shunichi Yokoyama

**Affiliations:** 1 Division of Clinical Application, Nanpuh Hospital, Kagoshima, Japan; 2 Gastroenterology, Nanpuh Hospital, Kagoshima, Japan; 3 Radiology, Nanpuh Hospital, Kagoshima, Japan; 4 Neurosurgery, Nanpuh Hospital, Kagoshima, Japan; Biomedical Research Foundation, UNITED STATES

## Abstract

A relationship between Alzheimer's disease (AD) and folate has been reported. Amyloid positron emission tomography (PET) is currently one of the most reliable biomarkers for AD. We investigated the correlation between serum folate levels and amyloid imaging to clarify whether serum folate could be a biomarker for AD. We also examined the usefulness of a combined assessment of serum folate levels and red blood cell hemoglobin content. Apolipoprotein E (APOE) gene polymorphisms were also assessed. Serum folate levels and hemoglobin content were evaluated by receiver operating characteristic analysis for their diagnostic capability as AD biomarkers relating to brain amyloid β accumulation. The area under the ROC curve (AUC) for serum folate was 0.136 (95% confidence interval [CI]: 0.000–0.312; p = 0.016). The AUC for hemoglobin content was 0.848 (95% CI: 0.661–1.000; p = 0.021). Therefore, the folate deficiency with low folate levels or the non-anaemia with high hemoglobin content levels were found to have a high probability of also testing positive for amyloid. Furthermore, eight patients were found to be folate deficiency and non-anaemia, those who were consist of 7 amyloid positive patients (87.5%), and only one of the amyloid negative patients (12.5%). These results suggest that a deficiency of serum folate and high hemoglobin levels may reflect an increased risk of amyloid β accumulation in the brain. Additionally, we demonstrated that these biomarkers could enhance the effectiveness of APOE as an AD biomarker. This study reveals that the combined assessment of serum folate levels and red blood cell hemoglobin content may be a useful biomarker for amyloid β accumulation in the brain. We also found that the combination of serum folate levels and hemoglobin content is a more specific and sensitive blood biomarker for AD than APOE or folate alone. These findings may be used to support clinical diagnosis of AD using a simple blood test.

## Introduction

The relationship between Alzheimer’s disease (AD) and decreased serum folate levels has been established [1]. It has been reported that DNA repair in nerve cells is inhibited by amyloid β-induced oxidative stress accompanied by folate deficiency [2]. However, the relationship between folate deficiency and the pathogenic mechanisms of AD has not been clearly elucidated.

The amyloid cascade hypothesis is a strong model for the pathogenesis of AD [3–5]. The accumulation of amyloid β in the brain begins more than 10 years before the appearance of dementia symptoms [6]. Amyloid imaging technology has been developed to detect brain amyloid β accumulation in the early stages of AD [7]. Amyloid positron emission tomography (PET) is one of strongest candidates for assessing AD biomarkers, based on the amyloid cascade hypothesis [8, 9].

In addition, it has been reported that the apolipoprotein E (APOE) gene polymorphism epsilon 4 (APOE- epsilon 4) is associated with familial and sporadic forms of AD [10]. Furthermore, carriers of APOE-epsilon 4, have been found to have greater deposits of amyloid β in the brain [11, 12]. However, there are a number of AD patients who do not carry APOE-epsilon 4, and the sensitivity of diagnosing AD according to the presence or absence of APOE-epsilon 4 is approximately 60% [13]. Therefore, when the presence or absence of APOE-epsilon 4 is used as a marker, a diagnosis of AD will be missed in 40% of patients. The consensus report released by the Ronald and Nancy Reagan Research Institute of the Alzheimer's Association et al. concluded that using the APOE-epsilon 4 allele of the APOE gene alone as a biomarker for AD should be avoided [14].

Therefore, new biomarkers for AD based on the amyloid hypothesis are needed. We investigated the correlation between serum folate levels and amyloid imaging to determine whether serum folate levels could be a biomarker for the early detection of AD.

Additionally, a close correlation has been reported between the risk of mild cognitive impairment (MCI) and the hemoglobin content of red blood cells [15]. We examined the significance of red blood cell hemoglobin content in addition to serum folate levels as a biomarker of brain amyloid β accumulation. Furthermore, we evaluated whether their combined assessment improves the accuracy of using APOE gene polymorphisms as a biomarker for AD.

## Material and methods

The study was approved by the Ethics Committee of Nanpuh Hospital, Kagoshima Kyosaikai, Public Interest Inc. Association, Japan. Clinical examinations were performed according to the principles of the Declaration of Helsinki. Research content was obtained in writing. In this study, the patients who were diagnosed with dementia in outpatient visits at Nanpuh Hospital were targeted as participants. We recruited without restrictions on sex and age. The research started on February 15, 2012. The 17 participants (7 male and 10 female) in this study underwent amyloid PET and assessment of serum folate, red blood cell hemoglobin content, and APOE gene polymorphisms in our outpatient clinic. The mean age of the participants was 77.1±6.5 years (mean±standard deviation (SD); range, 68–89 years). As a diagnostic criterion for dementia, AD was diagnosed based on the Diagnostic and Statistical Manual of Mental Disorders, Fourth Edition [16]. MCI was diagnosed according to the criteria of Petersen *et al* [17].

PET was performed using a Discovery ST Elite PET scanner (General Electric Co., CT, USA). The academic use of ^11^C-Pittsburgh compound B (^11^C-PiB) was permitted by Dr. Chester A. Mathis, Department of Radiology, University of Pittsburgh Medical Center PET Facility, University of Pittsburgh School of Medicine (Pittsburgh, PA, USA).

The dose of ^11^C-PiB was 555±185 MBq. The PET and magnetic resonance image volumes were co-registered. The regions of interest (ROI) were defined according to previous reports [18]. Amyloid state in the whole brain was visualized using by amyloid PET, based on accumulations in the precuneus, posterior cingulate gyrus, frontal cortex, lateral temporal cortex, lateral parietal cortex, and striatum. Stronger cortical retention than white matter in at least one cortex was assessed as amyloid positive and weaker cortical retention than white matter was assessed as amyloid negative. The assessments were performed by radiologists who were blinded to the clinical data.

Serum folate levels were measured by a chemiluminescent enzyme immunoassay method using a UniCel DxI 800 Access Immunoassay System (Beckman Coulter Inc., CA, USA) in accordance with the manufacturer's instructions. In the sensitivity test, when measuring the control serum at the known concentration (1.5 to 2.5 ng / mL), the measured value is within ± 10%. When measuring the same sample three times, the coefficient of variation of the measured value was within 7%. Hemoglobin content was measured using a XE-5000 Hematology Analyzer (Sysmex, Co., Hyogo, Japan) in accordance with the manufacturer's instructions. APOE gene polymorphism analysis was performed using an invader method and a CytoFluor Series 4000 Fluorescence and Bioluminescence Reader (Applied Biosystems Co., MA, USA) in accordance with the manufacturer's instructions.

A receiver operating characteristic (ROC) curve was constructed to evaluate the diagnostic performance of serum folate levels or hemoglobin content in differentiating between amyloid positive and negative patients.

The statistical difference in the prevalence of APOE-epsilon 4 between the amyloid positive and negative patients was analyzed using the Chi-square test. Data were analyzed by using SPSS Version 23 (IBM Co., NY, USA). A value of P < 0.05 indicated a statistically significant difference.

## Results

Table 1 shows the gender, clinical diagnosis, age, Mini-Mental State Examination (MMSE) score, serum folate levels, hemoglobin content and APOE genotype of the amyloid positive and negative patients. Amyloid was positive in 11 and negative in six of the 17 participants. The 17 patients consisted of 10 AD and seven MCI patients, with positive amyloid in seven of the AD patients and four of the MCI patients. The amyloid positive and negative patients’ mean ages were 75.4±4.5 years (mean±SD; range, 68–81) and 80.2±8.7 years (mean±SD; range, 70–89), respectively. The mean MMSE scores were 23.2±2.9 points (mean±SD; range, 20–30). The amyloid positive and negative patients' mean MMSE scores were 22.6±2.5 points (mean±SD; range, 20–27) and 24.3±3.6 points (mean±SD; range, 20–30), respectively. The overall mean serum folate levels were 7.8±4.6 ng/mL (mean±SD; range, 3.1–16.8). Amyloid positive and negative patients’ mean serum folate levels were 6.2±3.6 ng/mL (mean±SD; range, 3.1–13.6) and 10.7±5.3 ng/mL (mean±SD; range, 5.2–16.8), respectively. The normal range of folate is 6–20 ng/mL and less than 5.9 ng/mL as indicative of possible folate deficiency [19]. In our participants, the mean hemoglobin content was 12.8±1.9 g/dL (mean±SD; range, 8.9–15.8). Amyloid positive and negative patients’ mean hemoglobin content were 13.6±1.5 g/dL (mean±SD; range, 10.9–15.8) and 11.4±1.8 g/dL (mean±SD; range, 8.9–13.8), respectively. The normal range of hemoglobin levels in males (15 years of age and above) is greater than equal to 13.0 g/dL as indicative of non-anaemia and less than 13.0 g/dL as indicative of anaemia. And, non-pregnant women (15 years of age and above) is greater than equal to 12.0 g/dL as indicative of non-anaemia and less than 12.0 g/dL as indicative of anaemia [20]. Of the 17 participants, nine carried the APOE-epsilon 4 allele and eight had other APOE alleles. In the 11 patients positive for amyloid by PET eight carried the APOE-epsilon 4 allele and three carried other alleles. In patients negative for amyloid one had the APOE-epsilon 4 allele and the other five did not (Table 1).

**Table 1:** Patient profiles.

		Amyloid PET ^a^		
		Positive (n = 11)	Negative (n = 6)	Total (n = 17)
Gender	Male/ Female	5/6	2/4	7/10
Diagnosis	AD ^b^/MCI ^c^	7/4	3/3	10/7
Age (years)	Mean ± SD ^d^ (Range)	75.4 ± 4.5 (68–81)	80.2 ± 8.7 (70–89)	77.1 ± 6.5 (68–89)
MMSE ^e^ (points)	Mean ± SD ^d^ (Range)	22.6 ± 2.5 (20–27)	24.3 ± 3.6 (20–30)	23.2 ± 2.9 (20–30)
Folate level (ng/mL)	Mean ± SD ^d^ (Range)	6.2 ± 3.6 (3.1–13.6)	10.7 ± 5.3 (5.2–16.8)	7.8 ± 4.6 (3.1–16.8)
Hemoglobin content (g/dL)	Mean ± SD ^d^ (Range)	13.6 ± 1.5 (10.9–15.8)	11.4 ± 1.8 (8.9–13.8)	12.8 ± 1.9 (8.9–15.8)
APOE ^f^	Epsilon 4/ Non-epsilon 4	8/3	1/5	9/8

^a^ PET, positron emission tomography;

^b^ AD, Alzheimer's disease;

^c^ MCI, mild cognitive impairment;

^d^ SD, standard deviation;

^e^ MMSE, Mini Mental State Examination;

^f^ APOE, apolipoprotein E

**Fig 1:**
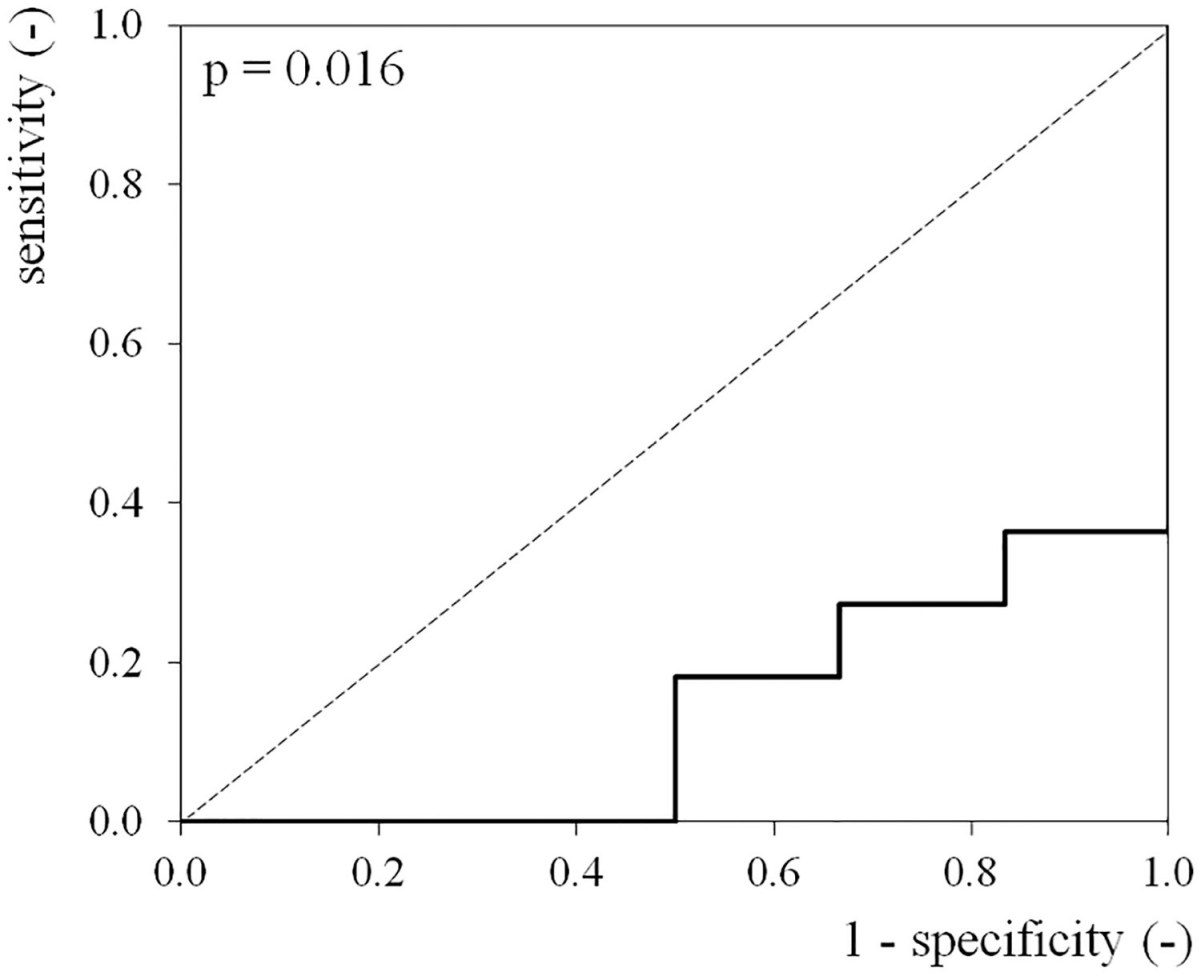
ROC curve of serum folate levels. An ROC curve was constructed to evaluate the diagnostic performance of serum folate levels in differentiating between amyloid positive patients and amyloid negative patients. The area under the ROC curve (AUC) for serum folate was 0.136 [95% CI: 0.000–0.312] (p = 0.016).

Serum folate levels and hemoglobin content were evaluated by ROC analysis for their diagnostic capability as AD biomarkers relating to brain amyloid β accumulation. The area under the ROC curve (AUC) for serum folate was 0.136 (95% confidence interval [CI]: 0.000–0.312; p = 0.016) (Fig 1). Therefore, the folate deficiency with low folate levels were found to have a high probability of also testing positive for amyloid.

**Fig 2:**
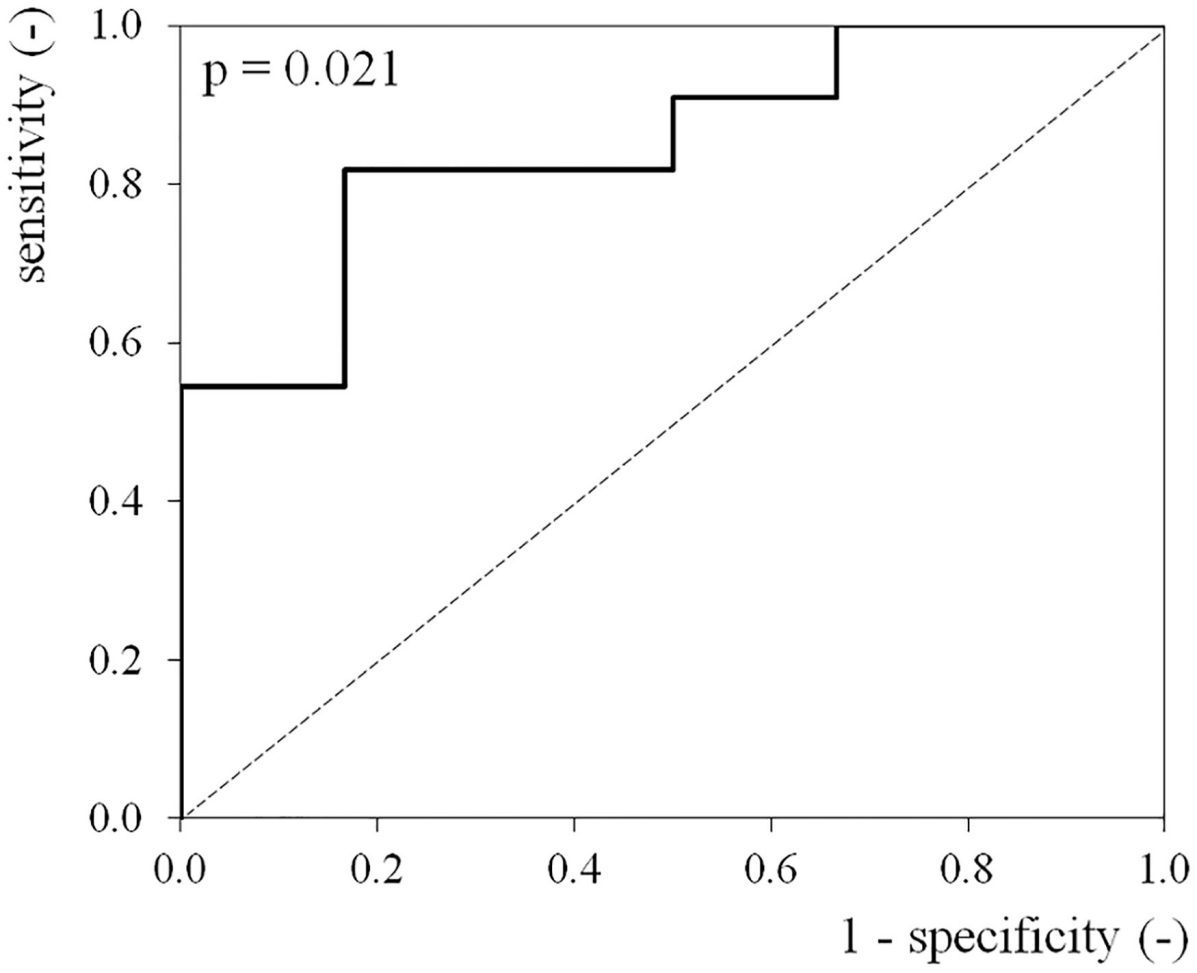
ROC curve of hemoglobin content. A ROC curve was constructed to evaluate the diagnostic performance of hemoglobin content in differentiating between amyloid positive patients and amyloid negative patients. The AUC for hemoglobin content was 0.848 [95% CI: 0.661–1.000] (p = 0.021).

In addition, we performed ROC analysis of hemoglobin content as an auxiliary diagnostic tool to serum folate levels. The AUC for hemoglobin content was 0.848 (95% CI: 0.661–1.000; p = 0.021) (Fig 2), showing high diagnostic performance. Therefore, the non-anaemia with high hemoglobin content levels were found to have a high probability of also testing positive for amyloid.

Folate deficiency and non-anaemia was seen in 7 of the 8 amyloid positive patients (87.5%), and only one of the amyloid negative patients (12.5%). Folate deficiency or non-anaemia was seen in 3 of the 5 amyloid positive patients (60.0%), and 2 of the amyloid negative patients (40.0%). Non-folate deficiency and anaemia was seen in 1 of the 4 amyloid positive patients (25.0%), and one of the amyloid negative patients (75.0%) (Table 2).

**Table 2:** Amyloid PET results of groups defined according to folate state and anaemia state.

	Amyloid PET positive patients	Amyloid PET negative patients	Total
Folate deficiency and non-anaemia	7	1	8
Folate deficiency or non-anaemia	3	2	5
The other cases	1	3	4
Total	11	6	17

**Fig 3:**
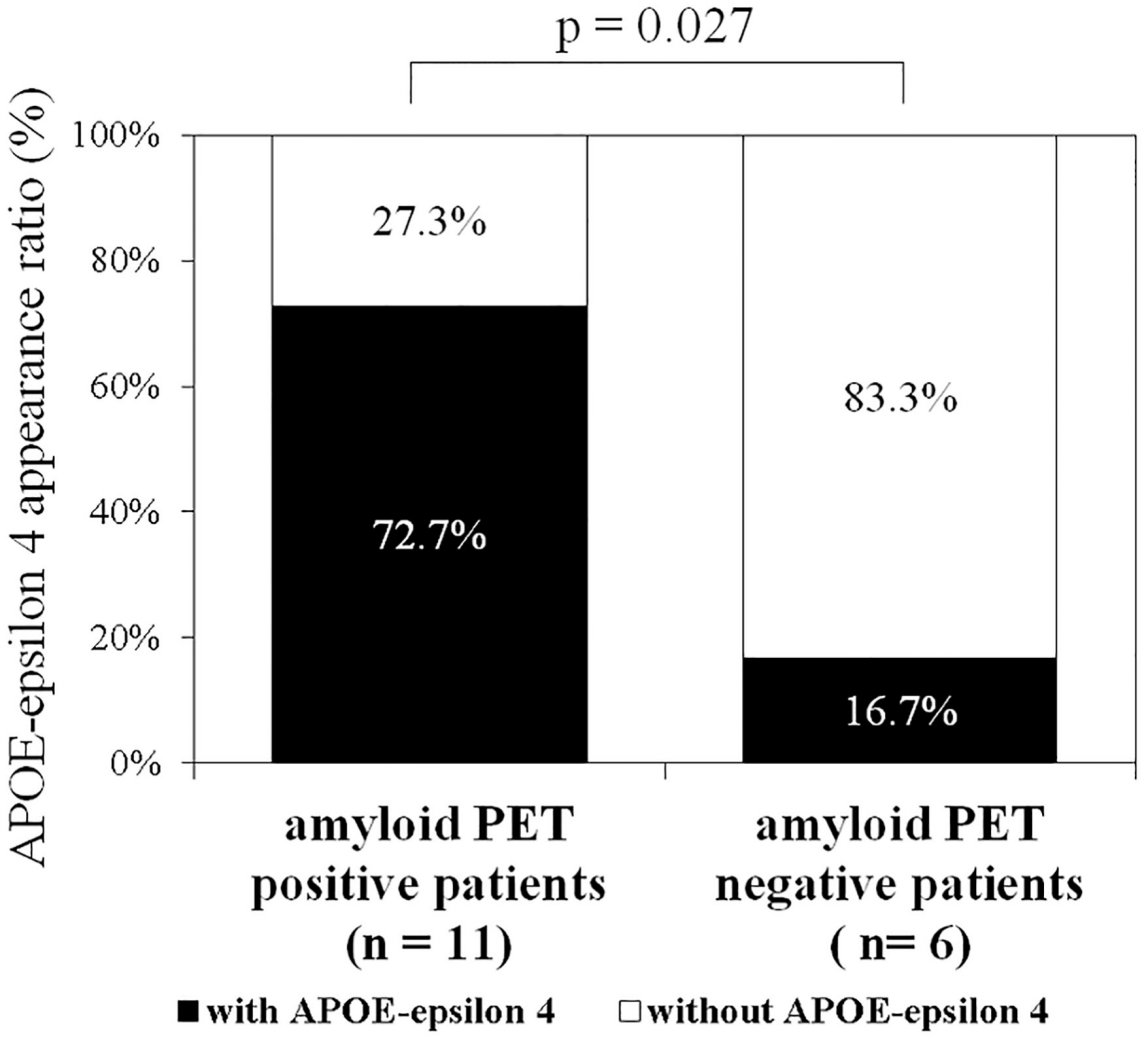
Prevalence of the APOE-epsilon 4 allele in amyloid positive and amyloid negative patients. Eight of the 11 (72.7%) amyloid positive patients also carried the APOE-epsilon 4 allele, with it occurring in only one of the six (16.7%) amyloid negative patients. The difference between the two groups was statistically significant (p = 0.027).

We also investigated the combination of folate levels, hemoglobin content, and the presence or absence of the APOE-epsilon 4 allele for its usefulness as a biomarker of AD relating to brain amyloid β accumulation. Fig 3 shows the APOE-epsilon 4 ratios in amyloid positive and amyloid negative patients. APOE-epsilon 4 appeared in eight of the 11 (72.7%) amyloid positive patients, and one of the six amyloid negative patients (16.7%). The difference of the incidence of the APOE-epsilon 4 allele between the two groups was statistically significant (p = 0.027) (Fig 3).

APOE-epsilon 4 carriers with folate deficiency and non-anaemia were defined as the APOE-epsilon 4 carriers plus α group, the APOE-epsilon 4 non-carriers with non-folate deficiency and anaemia were defined as the APOE-epsilon 4 non-carriers plus α group. All other cases were defined as the equivocal group. In the APOE-epsilon 4 carriers plus α group, all five patients were amyloid positive (100.0%). In the APOE-epsilon 4 non-carriers plus α group, all two patients were amyloid negative (100.0%) (Table 3).

**Table 3:** Amyloid PET results of groups defined according to APOE-epsilon 4 appearance, serum folate levels and hemoglobin content.

	Amyloid PET positive patients	Amyloid PET negative patients	Total
APOE-epsilon 4 carriers plus α group ^a^	5	0	5
APOE-epsilon 4 non-carriers plus α group ^b^	0	2	2
Equivocal group ^c^	6	4	10
Total	11	6	17

^a^ APOE-epsilon 4 carriers plus α group, APOE-epsilon 4 carriers with folate deficiency and non-anaemia;

^b^ APOE-epsilon 4 non-carriers plus α group, APOE-epsilon 4 non-carriers with non-folate deficiency and anaemia;

^c^ Equivocal group, All other cases

## Discussion

Amyloid β can induce oxidative stress and DNA damage in cultured neurons [21–23]. Both have been documented in neurons associated with amyloid β-containing plaques in the brains of AD patients [24, 25]. Oxidative stress and DNA damage induced by amyloid β play an important role in the pathogenesis of AD. Li W et al. reported that folate induces DNA methyltransferase (DNMT) activity, thereby attenuating amyloid β production [26].

Recent studies have revealed that DNMT activity is related to recent memory formation and as well as remote memory maintenance [27–28]. Moreover, Guo et al.’s study indicated that histone H3 hyperacetylation and DNMT-dependent hypomethylation mediate the activation of stress-related signaling pathways in SH-SY5Y cells, which leads to amyloid-β precursor protein, presenilin 1, and β-site APP-cleaving enzyme 1 gene expression increase, and thus to amyloid β overproduction [29]. In this study, we focused on hemoglobin. Jia-Ying et al. reported that hemoglobin binds to amyloid β, and enhances its aggregation [30]. High hemoglobin status leads to aggregation of amyloid β, suggesting that the risk of Alzheimer's disease is increased. Based on these data, we focused on folate as a biomarker for AD relating to amyloid β accumulation in the brain.

Recently, both cerebrospinal fluid (CSF) and amyloid PET studies have become widely used as reliable tools supporting the clinical diagnosis of AD, and are commonly used for assessment of the disease. Biomarkers of brain amyloid β protein deposition currently include low CSF amyloid β42 and positive PET amyloid imaging [31–32]. However, CSF collection imposes a large burden on the patient and amyloid PET assessment is costly. In addition, only a limited number of hospitals can perform these assessments. Therefore, this study focused on the development of biomarkers that can be readily assessed from blood samples. Blood biomarkers have the merit of also being less of a burden on patients and can be accessed through samples that would be routinely taken from patients anyway. APOE as a blood biomarker of AD is reliable but is not sufficiently specific or sensitive. Therefore, blood biomarkers that are more specific and sensitive than the classic biomarkers, including APOE, are required. The present study focused on evaluating the potential of serum folate levels or blood cell hemoglobin content alone as an AD biomarker, or in combination with APOE to enhance the accuracy of diagnosis. In our result, it showed that 87.5% patients were amyloid positive out of all the patients who were found to be folate deficiency and non-anaemia. Furthermore, all cases were positive for AD when tested with the combination of three biomarkers; folate levels, hemoglobin content, and APOE. Conversely, all of the cases that were negative for AD were also negative for all of the three biomarkers.

Based on these data, the present study revealed that the combination of serum folate levels and red blood cell hemoglobin content is more specific and sensitive as a blood biomarker for AD than APOE or folate alone. These results may be used to support clinical diagnosis using a simple and easy blood test. However, this study have limitations to prove the risk of AD in folate deficiency and anemia since the small number of patients who underwent amyloid PET scanning. In this study, both serum folate levels and red blood cell hemoglobin content are within the normal range in amyloid PET positive patients. For future development, we need a larger number of samples in order to establish a diagnostic approach including healthy controls as well.

In summary, the combined assessment of serum folate levels and blood cell hemoglobin content may be an accurate blood biomarker of amyloid β accumulation in the brain and may have the potential to compensate for the relative weakness of APOE as an AD biomarker.

## References

[1] Tian Tian, Bai Dong, Li Wen, Huang Guo-Wei, Liu Huan. Effects of Folic Acid on Secretases Involved in Aβ Deposition in APP/PS1 Mice. Nutrients. 2016 9 9; 2016 Sep; 8(9): 556. 10.3390/nu8090556 PMC5037541 27618097

[2] Kruman II, Kumaravel TS, Lohani A, Pedersen WA, Cutler RG, Kruman Y, et al Folic acid deficiency and homocysteine impair DNA repair in hippocampal neurons and sensitize them to amyloid toxicity in experimental models of Alzheimer's disease. J Neurosci. 2002 3 1; 22(5):1752–62. 11880504 10.1523/JNEUROSCI.22-05-01752.2002 PMC6758871

[3] Hardy JA, Higgins GA. Alzheimer's disease: the amyloid cascade hypothesis. Science. 1992 4 10; 256(5054):184–5. 1566067 10.1126/science.1566067

[4] Small DH, McLean CA. Alzheimer's disease and the amyloid beta protein: What is the role of amyloid? J Neurochem. 1999 8; 73(2):443–9. 10428038 10.1046/j.1471-4159.1999.0730443.x

[5] Jarrett JT, Lansbury PT Jr. Seeding "one-dimensional crystallization" of amyloid: a pathogenic mechanism in Alzheimer's disease and scrapie? Cell. 1993 6 18; 73(6):1055–8. 8513491 10.1016/0092-8674(93)90635-4

[6] Sperling RA, Aisen PS, Beckett LA, Bennett DA, Craft S, Fagan AM, et al Toward defining the preclinical stages of Alzheimer's disease: recommendations from the National Institute on Aging-Alzheimer's Association workgroups on diagnostic guidelines for Alzheimer's disease. Alzheimers Dement. 2011 5; 7(3):280–92. 10.1016/j.jalz.2011.03.003 21514248 PMC3220946

[7] Shimada H, Ataka S, Takeuchi J, Mori H, Wada Y, Watanabe Y. Pittsburgh compound B-negative dementia: a possibility of misdiagnosis of patients with non-alzheimer disease-type dementia as having AD. J Geriatr Psychiatry Neurol. 2011 9; 24(3):123–6. 10.1177/0891988711409410 21750305

[8] Albert MS, DeKosky ST, Dickson D, Dubois B, Feldman HH, Fox NC, et al The diagnosis of mild cognitive impairment due to Alzheimer's disease: recommendations from the National Institute on Aging-Alzheimer's Association workgroups on diagnostic guidelines for Alzheimer's disease. Alzheimers Dement. 2011 5; 7(3):270–9. 10.1016/j.jalz.2011.03.008 21514249 PMC3312027

[9] Klunk WE, Engler H, Nordberg A, Wang Y, Blomqvist G, Holt DP, et al Imaging brain amyloid in Alzheimer's disease with Pittsburgh Compound-B. Ann Neurol. 2004 3; 55(3):306–19. 10.1002/ana.20009 14991808

[10] Corder EH, Saunders AM, Strittmatter WJ, Schmechel DE, Gaskell PC, Small GW, et al Gene dose of apolipoprotein E type 4 allele and the risk of Alzheimer's disease in late onset families. Science. 1993 8 13; 261(5123):921–3. 8346443 10.1126/science.8346443

[11] O'Donnell HC, Rosand J, Knudsen KA, Furie KL, Segal AZ, Chiu RI, et al Apolipoprotein E genotype and the risk of recurrent lobar intracerebral hemorrhage. N Engl J Med. 2000 1 27; 342(4):240–5. 10.1056/NEJM200001273420403 10648765

[12] Reiman EM, Caselli RJ, Yun LS, Chen K, Bandy D, Minoshima S, et al Preclinical evidence of Alzheimer's disease in persons homozygous for the epsilon 4 allele for apolipoprotein E. N Engl J Med. 1996 3 21; 334(12):752–8. 10.1056/NEJM199603213341202 8592548

[13] Higuchi M., Arai H., Okamura N., Tashiro M., Matsui T., Higuchi S., et al Apolipoprotein E and Alzheimer's Disease Therapeutic Implications. 1999 Mol Diag Ther (1999) 11: 411.

[14] The Ronald and Nancy Reagan Research Institute of the Alzheimer's Association and, National Institute on Aging Working Group. Consensus report of the Working Group on: "Molecular and Biochemical Markers of Alzheimer's Disease". The Ronald and Nancy Reagan Research Institute of the Alzheimer's Association and the National Institute on Aging Working Group. Neurobiol Aging. 1998 Mar-Apr; 19(2):109–16. 9558143

[15] Dlugaj M, Winkler A, Weimar C, Durig J, Broecker-Preuss M, Dragano N, et al Anemia and Mild Cognitive Impairment in the German General Population. J Alzheimers Dis. 2015; 49(4):1031–42. 10.3233/JAD-150434 26599053

[16] American Psychiatric Association. Diagnostic and Statistical Manual of Mental Disorders, 4th edn Washington, DC: American Psychiatric Association, 1994.

[17] Petersen RC, Smith GE, Waring SC, Ivnik RJ, Tangalos EG, Kokmen E. Mild cognitive impairment: clinical characterization and outcome. Arch Neurol. 1999 3; 56(3):303–8. 10190820 10.1001/archneur.56.3.303

[18] McNamee RL, Yee SH, Price JC, Klunk WE, Rosario B, Weissfeld L, et al Consideration of optimal time window for Pittsburgh compound B PET summed uptake measurements. J Nucl Med. 2009 3; 50(3):348–55. 10.2967/jnumed.108.057612 19223409 PMC2694747

[19] WHO; Serum and red blood cell folate concentrations for assessing folate status in population, 2012

[20] WHO; Hemoglobin concentrations for the diagnosis of anaemia and assessment of severity, 2011

[21] Loo DT, Copani A, Pike CJ, Whittemore ER, Walencewicz AJ, Cotman CW. Apoptosis is induced by beta-amyloid in cultured central nervous system neurons. Proc Natl Acad Sci U S A. 1993 9 1; 90(17):7951–5. 8367446 10.1073/pnas.90.17.7951 PMC47265

[22] Mark RJ, Hensley K, Butterfield DA, Mattson MP. Amyloid beta-peptide impairs ion-motive ATPase activities: evidence for a role in loss of neuronal Ca2+ homeostasis and cell death. J Neurosci. 1995 9; 15(9):6239–49. 7666206 10.1523/JNEUROSCI.15-09-06239.1995 PMC6577674

[23] Mark RJ, Pang Z, Geddes JW, Uchida K, Mattson MP. Amyloid beta-peptide impairs glucose transport in hippocampal and cortical neurons: involvement of membrane lipid peroxidation. J Neurosci. 1997 2 1; 17(3):1046–54. 8994059 10.1523/JNEUROSCI.17-03-01046.1997 PMC6573165

[24] Mattson MP. Cellular actions of beta-amyloid precursor protein and its soluble and fibrillogenic derivatives. Physiol Rev. 1997 10; 77(4):1081–132. 9354812 10.1152/physrev.1997.77.4.1081

[25] Mattson MP. Apoptosis in neurodegenerative disorders. Nat Rev Mol Cell Biol. 2000 11; 1(2):120–9. 10.1038/35040009 11253364

[26] Li W, Jiang M, Zhao S, Liu H, Zhang X, Wilson JX, et al Folic Acid Inhibits Amyloid β-Peptide Production through Modulating DNA Methyltransferase Activity in N2a-APP Cells. Int J Mol Sci. 2015 10 20; 16(10):25002–13. 10.3390/ijms161025002 26492244 PMC4632786

[27] Heyward FD, Sweatt JD. DNA Methylation in Memory Formation: Emerging Insights. Neuroscientist. 2015 10; 21(5):475–89. 10.1177/1073858415579635 25832671 PMC4770785

[28] Bie B, Wu J, Yang H, Xu JJ, Brown DL, Naguib M. Epigenetic suppression of neuroligin 1 underlies amyloid-induced memory deficiency. Nat Neurosci. 2014 2; 17(2):223–31. 10.1038/nn.3618 24441681

[29] Guo X, Wu X, Ren L, Liu G, Li L. Epigenetic mechanisms of amyloid-β production in anisomycin-treated SH-SY5Y cells. Neuroscience. 2011 10 27; 194:272–81. 10.1016/j.neuroscience.2011.07.012 21843603

[30] Chuang JY, Lee CW, Shih YH, Yang T, Yu L, Kuo YM. Interactions between amyloid-β and hemoglobin: implications for amyloid plaque formation in Alzheimer's disease. PLoS One. 2012 3 6; 7(3):e33120 10.1371/journal.pone.0033120 22412990 PMC3295782

[31] McKhann GM, Knopman DS, Chertkow H, Hyman BT, Jack CR Jr, Kawas CH, et al The diagnosis of dementia due to Alzheimer's disease: recommendations from the National Institute on Aging-Alzheimer's Association workgroups on diagnostic guidelines for Alzheimer's disease. Alzheimers Dement. 2011 5; 7(3):263–9. 10.1016/j.jalz.2011.03.005 21514250 PMC3312024

[32] Albert MS, DeKosky ST, Dickson D, Dubois B, Feldman HH, Fox NC, et al The diagnosis of mild cognitive impairment due to Alzheimer's disease: recommendations from the National Institute on Aging-Alzheimer's Association workgroups on diagnostic guidelines for Alzheimer's disease. Alzheimers Dement. 2011 5; 7(3):270–9. 10.1016/j.jalz.2011.03.008 21514249 PMC3312027

